# Active Matrix Metalloproteinase-8 (aMMP-8) Versus Total MMP-8 in Periodontal and Peri-Implant Disease Point-of-Care Diagnostics

**DOI:** 10.3390/biomedicines11112885

**Published:** 2023-10-25

**Authors:** Ismo T. Räisänen, Nur Rahman Ahmad Seno Aji, Dimitra Sakellari, Andreas Grigoriadis, Iina Rantala, Tommi Pätilä, Pia Heikkilä, Shipra Gupta, Timo Sorsa

**Affiliations:** 1 Department of Oral and Maxillofacial Diseases, Head and Neck Center, University of Helsinki and Helsinki University Hospital, 00290 Helsinki, Finland; 2 Department of Periodontics, Faculty of Dentistry, Universitas Gadjah Mada, Jalan Denta No.1 Sekip Utara, Sleman, Yogyakarta 55281, Indonesia; 3 Department of Preventive Dentistry, Periodontology and Implant Biology, Faculty of Health Sciences, Dental School, Aristotle University of Thessaloniki, 541 24 Thessaloniki, Greece; 4 Dental Sector, 424 General Military Training Hospital, 564 29 Thessaloniki, Greece; 5 Department of Pediatric Surgery, New Children’s Hospital, University of Helsinki and Helsinki University Hospital, 00290 Helsinki, Finland; 6 Oral Health Sciences Centre, Post Graduate Institute of Medical Education & Research (PGIMER), Chandigarh 160012, India; 7 Department of Oral Diseases, Karolinska Institutet, 171 77 Stockholm, Sweden

**Keywords:** aMMP-8, periodontitis, peri-implantitis, matrix metalloproteinase, biomarker, point-of-care diagnostics, oral fluid

## Abstract

Active matrix metalloproteinase-8 (aMMP-8) is a promising biomarker candidate for the modern periodontal and peri-implant disease diagnostics utilizing the chairside/point-of-care oral fluid technologies. These rapid biomarker analysis technologies utilize gingival crevicular fluid (GCF), peri-implant sulcular fluid (PISF), or mouth rinse as the oral fluid matrices that can be collected patient-friendly and non-invasively without causing bacteremia. aMMP-8, but not total or latent proMMP-8, has been shown to be a relevant biomarker to be implemented to the latest 2017 classification system of periodontitis and peri-implantitis. Thus, aMMP-8 point-of-care-testing (POCT)—but not total or latent proMMP-8—can be conveniently used as an adjunctive and preventive diagnostic tool to identify and screen the developing and ongoing periodontal and peri-implant breakdown and disease as well as predict its episodic progression. Similarly, aMMP-8 POCT provides an important tool to monitor the treatment effect of these diseases, but also other diseases such as head and neck cancer, where it can identify and predict the rapid tissue destructive oral side-effects during and after the radiotherapy. Additionally, recent studies support aMMP-8 POCT benefitting the identification of periodontitis and diabetes as the escalating risk diseases for COVID-19 infection. Overall, aMMP-8 POCT has launched a new clinical field in oral medicine and dentistry, i.e., oral clinical chemistry.

## 1. Introduction

Periodontitis and peri-implantitis are chronic multifactorial oral diseases with infectious and inflammatory elements where the homeostasis of the complex interactions between polymicrobial biofilm and host inflammatory and immune response eventually is disrupted, leading to the destruction of the tissues that support teeth/dental implants and, finally, to the loss of teeth/dental implants [1,2]. Periodontitis is a global, wide-spread disease and its severe form has been estimated to affect globally at least 10%, i.e., almost 800 million people [3]. The prevalence of peri-implantitis is also common among patients with dental implants, and its prevalence has been estimated in the literature to be 4.7–45% at the patient level and 3.6–22.1% at the dental implant level [4].

The diagnosis of periodontitis and peri-implantitis typically relies on the clinical and radiographical evaluations of the disease manifestations (deepened probing depths, clinical attachment loss, bleeding on probing, etc.) [5,6,7]. This traditional diagnostic approach determines the severity and extent of the past periodontal/peri-implant tissue destruction and attachment loss, but it is not accurate enough to reliably detect the early and initial stages of the periodontal disease or peri-implantitis, nor their current or future disease activities [5,6]. Hence to overcome this limitation, biological biomarkers in oral fluids (gingival crevicular fluid (GCF), peri-implant sulcular fluid (PISF), saliva and mouth rinse) have been researched substantially in order to better utilize them to increase the accuracy of the early diagnostics, as well as for risk assessments of periodontal disease and peri-implantitis and their future progression [8,9,10,11,12].

Oral fluids can be easily collected non-invasively without the risk of bacteremia for monitoring biochemical processes associated with periodontitis and peri-implantitis reflected in the levels of biomarkers [13]. Collecting oral fluids is rarely unpleasant to patients. So far, among an extensive number of potential biomarkers that the periodontal and peri-implant biomarker research has investigated active matrix metalloproteinase-8 (aMMP-8), has been one of the most successful for periodontitis and peri-implantitis [8,9,10,11,12]. aMMP-8 (also known as active collagenase-2 or neutrophil collagenase) belongs to the family of tissue destructive enzymes related to periodontitis and peri-implantitis, where the elevation of activated MMP-8 to pathological levels plays an important role in the initiation and progression of the tissue destructive disease and related attachment loss [14,15,16].

## 2. Active and Total MMP-8 Should Be Distinguished in Periodontal and Peri-Implant Diagnostics

**Figure 1:**
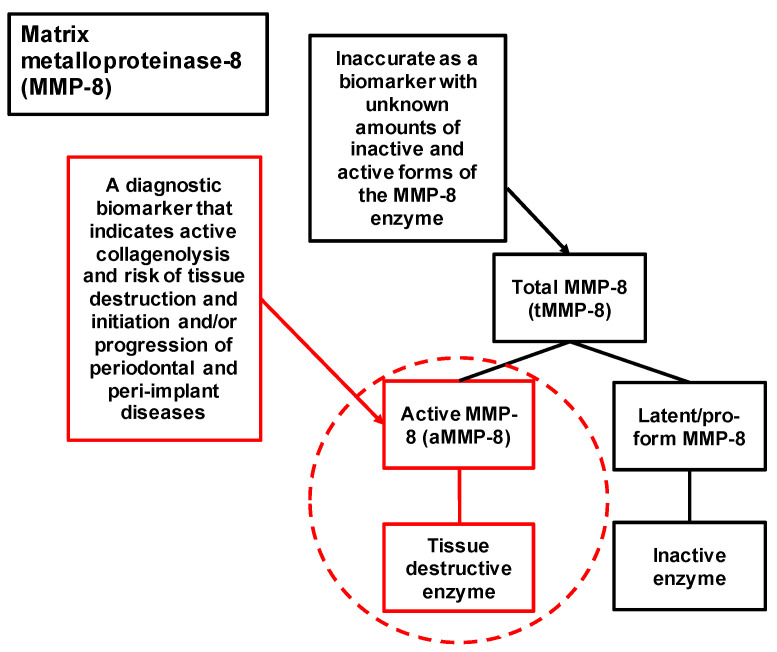
Graphical conclusion: mouth rinse, gingival crevicular fluid (GCF) and peri-implant sulcular fluid (PISF) aMMP-8 levels utilized as the biomarker of periodontitis and peri-implantitis.

The excellent and concise mini-review entitled “Matrix Metalloproteinases in Oral Health-Special Attention on MMP-8” was recently published by Atanasova et al. [17]. Their comprehensive and updated mini-review deals mainly with MMP-8 that corresponds to total MMP-8 or latent proMMP-8 [17]. However, it is vital to differentiate total MMP-8 (tMMP-8) or latent proMMP-8 and active MMP-8 (aMMP-8) from each other in the oral health biomarker research because there is a clear difference between them (Figure 1) [14,15,16]. Latent proMMP-8 is enzymatically inactive, thus not collagenolytic [14,15,16]. To be catalytically competent, i.e., collagenolytic and proteolytic, latent proMMP-8 requires activation by other host and microbial proteases and/or by reactive oxygen radicals to aMMP-8, which can process and degrade collagens, extracellular matrix components, non-matrix bioactive molecules, complement components, serpins and insulin-receptor [12,16]. Regarding the irreversible tissue destruction pathogenesis of oral diseases, periodontitis and peri-implantitis, as well as systemic diseases such as diabetes, sepsis, meningitis, kidney diseases, pancreatitis, chronic obstructive pulmonary disease (COPD), cardiovascular diseases (CVD) and cancers; the key collagenolytic MMP has been demonstrated to be aMMP-8 (i.e., collagenase-2/neutrophil collagenase) [18,19,20,21,22,23,24,25,26,27,28,29,30,31,32]. aMMP-8 is catalytically competent and tissue destructive enzyme in the clinically active disease sites and progressive lesions in these oral and systemic diseases [16].

Diagnostic studies and technologies attempting to utilize total MMP-8 as a diagnostic biomarker for periodontitis and peri-implantitis have shown contrasting outcomes as some of the studies have revealed relatively good diagnostic ability of total MMP-8 to discriminate periodontal and peri-implant health and disease, while many others have clearly shown the opposite [33,34,35,36,37,38,39,40,41,42]. So far, only studies on aMMP-8 oral fluid diagnostic biomarker analysis with different and independent techniques and technologies have repeatedly and consistently proved to be successful [18,19,20,21,22,23,24,25,26,27,28,29,30,31,32]. Recent studies have shown aMMP-8 to be an applicable biomarker to be implemented in the new 2017 classification systems’ stages and grades of periodontitis and peri-implantitis assessment [26,27,28,29,30,31,32,43,44]. In contrast, total MMP-8 (ELISA, enzyme-linked immunosorbent assay) has not been able, to the authors knowledge, to differentiate different stages of periodontitis, whereas aMMP-8 has [26,27,28,36,37,38,40,42].

**Figure 2:**
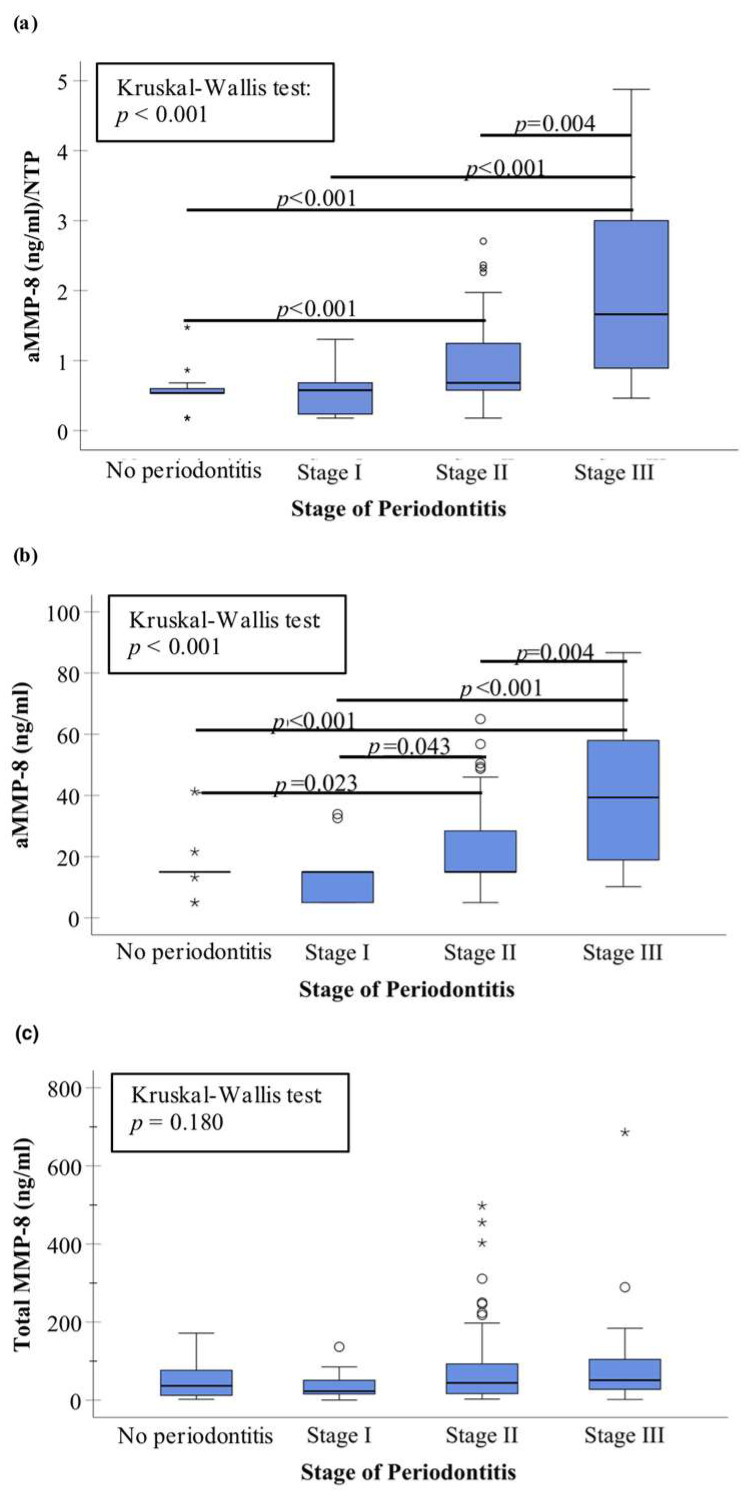
The association between the stage of periodontitis and (**a**) active matrix metalloproteinase (aMMP)-8 PoC test per the number of teeth present (aMMP-8/NTP), (**b**) aMMP-8 PoC test, and (**c**) total MMP-8 (ELISA) in 150 Greek adults as described in Sorsa et al. (2020) and Gupta et al. (2023) [26,42]. Kruskal–Wallis test was significant (*p* < 0.001) for aMMP-8 and aMMP-8/NTP, but not for total MMP-8 (*p* = 0.180). All significant (*p* < 0.05) pairwise post hoc comparisons (Dunn–Bonferroni test) are marked in the plots. The asterisk (*) indicates an extreme outlier (1.5× interquartile range) in the data. (**b**,**c**) reproduced from Sorsa et al. (2020) and Gupta et al. (2023), respectively [26,42] under the terms and conditions of the Creative Commons Attribution (CC BY) license https://creativecommons.org/licenses/by/4.0/, accessed on 28 August 2023.

**Figure 3:**
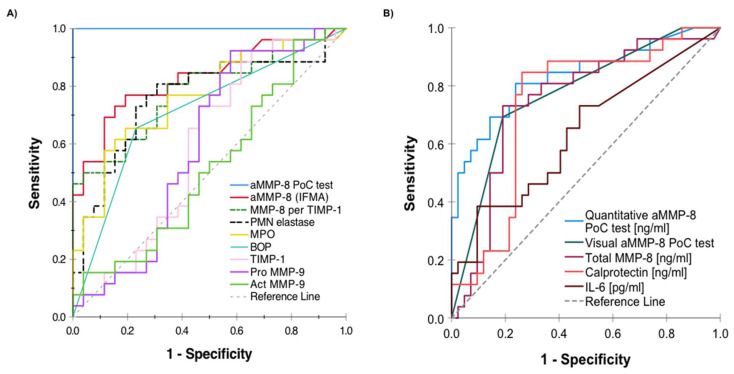
Receiver operating characteristic (ROC) analysis presenting the diagnostic ability of aMMP-8 versus other biomarker candidates to discriminate peri-implantitis from healthy implant among (**A**) 26 peri-implantitis and 26 healthy dental implant patients as described in Lähteenmäki et al. (2020) [45] and (**B**) 26 peri-implantitis and 42 healthy dental implants as described in Lähteenmäki et al. (2022) [44]. Performance of a biomarker in ROC analysis is measured by the area under the individual ROC curves. (**A**,**B**) reproduced from Lähteenmäki et al. (2020) and Lähteenmäki et al. (2022) [44,45] under the terms and conditions of the Creative Commons Attribution (CC BY) license (https://creativecommons.org/licenses/by/4.0/, accessed on 28 August 2023).

Recent studies have shown the association between aMMP-8 and stage of periodontitis (Figure 2b), while total MMP-8 (ELISA) was not associated with stage of periodontitis (Figure 2c). Thus, total MMP-8 containing both latent proMMP-8 and active MMP-8 seems too prone to inaccuracies because of latent proMMP-8 and is not accurate enough for periodontal disease diagnostics, whereas measuring aMMP-8 levels leads to more accurate presentation of the collagenolytic tissue destruction in the clinically active disease sites and progressive lesions (Figure 1). Moreover, aMMP-8 can be analyzed in peri-implantitis diagnostics by traditional laboratory method immunofluorometric assay (IFMA) and modern rapid point-of-care/chairside diagnostics (aMMP-8 POCT), and both methods have shown aMMP-8 to be more precise (in terms of the area under the receiver operating curve (ROC)) than the other tested biomarkers (total MMP-8, polymorphonuclear (PMN) elastase, myeloperoxidase (MPO), tissue inhibitor of MMP (TIMP)-1, proMMP-9, aMMP-9) and bleeding on probing (BOP) (Figure 3, Table 1).

## 3. aMMP-8 or aMMP-8 per the Number of Teeth Present (aMMP-8/NTP)

**Figure 4:**
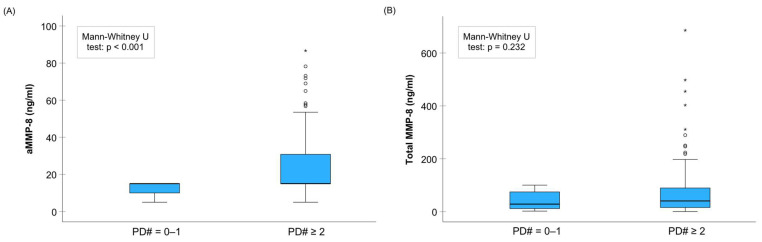
(**A**) Active matrix metalloproteinase (aMMP)-8 and (**B**) total MMP-8 levels in 150 Greek adults with 0–1 and ≥2 periodontal probing depths of ≥4 mm, aMMP-8 by point-of-care testing and total MMP-8 by ELISA were measured as described in Sorsa et al. (2020) and Gupta et al. (2023) [26,42]. The asterisk (*) indicates an extreme outlier (1.5× interquartile range) in the data.

A recent study suggested using aMMP-8 per the number of teeth present (aMMP-8/NTP) in periodontal disease diagnostics to measure the average aMMP-8 levels per tooth [27]. This opinion article presents here similar findings that aMMP-8/NTP is associated with stage of periodontitis (*p* < 0.001; Kruskal–Wallis test), which is in agreement with and further extends the previous findings (Figure 2a). Deng et al. (2021) found that aMMP-8 with NTP adjustment could perform better than without the adjustment, while the results presented here suggest the opposite [27]. Pairwise comparisons performed by a Dunn–Bonferroni test revealed significant differences between no periodontitis group against stage II and stage III of periodontitis groups, as well as stage III of periodontitis group against stage I and stage II of periodontitis groups (Figure 2a). The same significant pairwise differences, but also a significant difference between stage I and II of periodontitis groups in the same sample of Greek adults, were recently reported by Sorsa et al. (2020) for aMMP-8 without averaging its levels for the number of teeth present (Figure 2b) [26]. A Dunn–Bonferroni test showed no significant pairwise differences in total MMP-8 levels between the stages of periodontitis (Figure 2c). Here, the study sample consisted of 150 Greek adult patients (age 25–78 years) of the Department of Periodontology, Dental School, Aristotle University, Thessaloniki, Greece, and the Periodontal Department of 424 General Army Hospital, Thessaloniki, Greece, underwent a full-mouth periodontal examination and they provided a mouth rinse sample in order to analyze the aMMP-8 levels by a chairside/point of care immunotest testing equipment combined with digital reader as described previously [26,42,47]. Briefly, there was a 30 s pre-rinsing with tap water and a one minute wait after the pre-rinse before collecting the mouth rinse sample after a 30 s of rinsing with 5 mL of test solution. Three drops of the sample solution were used in the test system, and the result was read on the digital reader within 5–6 min. Thus, the aMMP-8 levels were analyzed by a similar aMMP-8 point of care testing equipment as Deng et al. (2021) in their aMMP-8/NTP analysis [27]. Similarly, patients’ periodontal disease diagnosing was conducted in both studies according to the 2017 classification system [6,26,27]. However, one explanation to the difference in aMMP-8 and aMMP-8/NTP performance for periodontitis diagnostics between the Hong Kong (Deng et al. 2021) and the Greek samples (the present study) may be the differences in periodontal status of participants as suggested by Sorsa et al. (2021) [26,27]. For example, participants in the Hong Kong sample seemed to have much lower levels of gingival inflammation and potentially better oral health behavior in some way than the Greek participants in the present study, as they had much lower bleeding on probing levels. That may naturally decrease the aMMP-8 levels in the Hong Kong sample compared with the Greek sample in the present study, which may affect to some extent to the aMMP-8 results. Additionally, aMMP-8 levels seem to be more accurate than total MMP-8 levels to detect periodontal pocketing (Figure 4). There was a significant association between aMMP-8 levels and patients having at least two ≥4 mm periodontal pockets in the Greek sample, while total MMP-8 levels were not associated with periodontal pocketing. This further extends previous findings [26,42]. Nevertheless, more studies are required to extend our knowledge in this regard. Here in this study, the statistical analyses of Figure 2a were performed with the SPSS Statistics 29.0.0.0. Statistical Software Package (SPSS Inc., IBM, Chicago, IL, USA). Data analysis was performed and the figure plotted with the statistical software.

## 4. Mouth Rinse Versus Saliva in Periodontal Disease Diagnostics

Mouth rinse is generally a collection of whole mouth (i.e., all teeth) gingival crevicular fluid, and can be used to analyze the whole mouth aMMP-8 levels derived from neutrophils and thus represents basically the whole mouth periodontium’s neutrophil collagenase activity from the sites of periodontal inflammation [48,49]. Taking into account the number of teeth present (i.e., previous tooth loss) may be a potentially beneficial variable to be combined with aMMP-8 levels in periodontal disease diagnostics. On the other hand, mouth rinse and saliva are not the same oral fluids, as saliva contains not only GCF but also many other products and different components from the salivary glands and other sources that may also confound aMMP-8 analysis. For example, collecting mouth rinse has the advantage to minimize some possible interfering components present in saliva, such as tissue inhibitors of matrix metalloproteinases (TIMPs) that are protease inhibitors decreasing collagenase activity [48]. This has been demonstrated in recent studies that have shown that mouth rinse is more precise diagnostic oral fluid matrix compared with saliva and should be preferred in aMMP-8 studies [26,30,50,51]. Furthermore, mouth rinse collection with standardized test liquid volume has a clear advantage against saliva collection with variable salivary flow rates and volumes that may have an effect on the biomarker concentrations in the final analysis. That way, patients can be better standardized for more accurate analysis, and the challenge with, for example, patients with xerostomia and hyposalivation can be minimized [46].

## 5. Conclusions

It is important to note that aMMP-8 has not only proven to a useful real-time on-line alarm of initial periodontitis in adolescents, but it has also benefits to a great extent in monitoring the outcomes of periodontal and peri-implant treatment modalities [45,52]. Thus, aMMP-8 POCT makes the invisible visible in the periodontal and peri-implant tissues, whereas traditional diagnostic methods mainly detect the past attachment loss in those tissues and provide much less precise prediction of the current disease activity and the progression of attachment loss. Previous studies have also found increased MMP-8 (and MMP-9) levels and less favorable healing process in the gingival tissues of patients with chronic periodontitis and diabetes compared with periodontitis patients without diabetes [53]. In this regard, aMMP-8 has been found to successfully identify and screen periodontitis together with diabetes as the escalating risk disease(s) for COVID-19 infection [44,54,55]. Periodontitis has the potential to increase the odds of COVID-19 infection, while compromised gum health and periodontitis may increase the odds of COVID-19 related complications such as hospital admissions and COVID-19 pneumonia, as well as mortality [54,56,57]. Additionally, aMMP-8 POCT can be and has been successfully used for monitoring the tissue destructive oral side effects resulting from the radiotherapy of head and neck cancers, and to identify the increased vulnerability of further periodontal tissue destruction [58,59]. Here, early identification of elevated aMMP-8 levels and periodontitis may aid in targeting patients requiring preventive measures and instituting early treatment.

Finally, oral fluid point-of-care diagnostic technologies utilizing periodontitis and peri-implantitis biomarkers such as aMMP-8 can benefit in the identification of patients at risk or undiagnosed with these diseases not only at the dentist’s office, but also in the hands of medical professionals, who can refer them to a dentist for further examination of their need of treatment. Point of care testing requires little dental expertise, and can be as simple to use as the classical pregnancy tests and COVID-19 tests are. Therefore, the testing could also be a useful as a teledentistry tool for targeted health promotion purposes that could be performed by a trained personnel or even people themselves in places and situations, for example, far away from a dentist or with people having difficulties to go and visit dentist, such as people in care homes. Overall, aMMP-8 has created a new clinical field in oral medicine and dentistry, namely the oral clinical chemistry that can benefit oral healthcare professionals towards more accurate and timely diagnostics, disease prevention, and monitoring of treatment outcomes.

## Figures and Tables

**Table 1:** Receiver operating characteristic (ROC) analysis and the area under the ROC curve (AUC) with 95% confidence interval illustrating the diagnostic ability of aMMP-8 versus other biomarker candidates to discriminate peri-implantitis from a healthy implant as described in Lähteenmäki et al. (2020) [45] (26 peri-implant and 26 healthy dental implant patients) and as described in Lähteenmäki et al. (2022) [44] (26 peri-implant and 42 healthy dental implant patients). The closer AUC value is to 1.0, the better the biomarker is to discriminate peri-implantitis from a healthy implant. Table is reproduced from Lähteenmäki et al. (2020) and Lähteenmäki et al. (2022) [44,45] under the terms and conditions of the Creative Commons Attribution (CC BY) license (https://creativecommons.org/licenses/by/4.0/, accessed on 28 August 2023).

Biomarkers	AUC (95% Confidence Interval)	*p*-Value	Study
aMMP-8 PoC test (visual)	1.000 (1.000–1.000)	<0.001	Lähteenmäki et al. (2020) [46]
aMMP-8 (IFMA)	0.829 (0.715–0.943)	<0.001	Lähteenmäki et al. (2020) [46]
MMP-8 per TIMP-1	0.787 (0.663–0.911)	<0.001	Lähteenmäki et al. (2020) [46]
PMN Elastase	0.765 (0.630–0.900)	0.001	Lähteenmäki et al. (2020) [46]
MPO	0.763 (0.632–0.894)	0.001	Lähteenmäki et al. (2020) [46]
BOP	0.712 (0.568–0.855)	0.009	Lähteenmäki et al. (2020) [46]
TIMP-1	0.593 (0.434–0.753)	0.249	Lähteenmäki et al. (2020) [46]
Pro MMP-9	0.598 (0.437–0.758)	0.227	Lähteenmäki et al. (2020) [46]
Active MMP-9	0.518 (0.358–0.677)	0.826	Lähteenmäki et al. (2020) [46]
aMMP-8 PoC test (quantitative)	0.833 (0.728–0.938)	<0.001	Lähteenmäki et al. (2022) [44]
aMMP-8 PoC test (visual)	0.773 (0.657–0.888)	<0.001	Lähteenmäki et al. (2022) [44]
Total MMP-8	0.750 (0.627–0.872)	0.001	Lähteenmäki et al. (2022) [44]
Calprotectin	0.736 (0.611–0.861)	0.001	Lähteenmäki et al. (2022) [44]
IL-6	0.637 (0.498–0.776)	0.059	Lähteenmäki et al. (2022) [44]

## Data Availability

The data that support the findings of this study are available on reasonable request from the corresponding author. The data are not publicly available due to privacy and ethical restrictions.
